# Expanded molecular detection of *MPL* codon p.W515 and p.S505N mutations in myeloproliferative neoplasms

**DOI:** 10.1002/jcla.24992

**Published:** 2023-12-07

**Authors:** Eric W. Miller, Celeste M. Lamberson, Ratilal R. Akabari, Michel R. Nasr, Steven M. Sperber

**Affiliations:** ^1^ Department of Pathology SUNY Upstate Medical University Syracuse New York USA

**Keywords:** MPL, W515, S505N, myeloproliferative

## Abstract

**Background:**

Patients negative for the *JAK2* p.V617F somatic variant are frequently reflexed to testing for *MPL* exon 10 variants. Detection of these variants via multiplexed allele‐specific PCR followed by fragment analysis has been previously published. The present study builds on this concept by improving the detection of the p.W515A variant, adding a second allele‐specific primer to detect the p.W515R variant, and incorporating an improved primer for p.S505N detection.

**Methods:**

The W515 amplification employs 5′‐labeled allele‐specific forward primers to detect p.W515K, p.W515L, p.W515R, and p.W515A. The p.S505N amplification includes an allele‐specific reverse primer with a tail extension. Fragments were subject to capillary electrophoresis on an ABI 3500 Genetic Analyzer and analyzed using GeneMapper 6.0 (Thermo Fisher Scientific).

**Results:**

Thirty *MPL‐*negative and 13 *MPL‐*positive samples previously tested by a reference laboratory were tested with the *MPL* LDT. Results were 100% concordant. The *MPL* LDT has a limit of detection of at least 5% VAF for the p.W515 variants and 10% VAF for the p.S505N variant.

**Conclusion:**

Current *MPL* assays are predominantly focused on p.W515L/K and p.S505N mutations. We have engineered an *MPL* test for detecting p.W515A/L/K/R and p.S505N variants, thereby increasing the diagnostic yield with little additional expense or technician time.

## Graphical abstract

*MPL* codon p.W515 and p.S505N variants are associated with essential thrombocythemia (ET) and myelofibrosis (MF) in *JAK2* p.V617F‐negative patients. This laboratory‐developed test allows for detecting the four most common p.W515 variants in conjunction with the p.S505N variant via allele‐specific PCR and subsequent fragment analysis. The p.W515 variants are detected using allele‐specific, 5′‐fluorochrome labeled primers. In contrast, the p.S505N variant is detected using an unlabeled, allele‐specific reverse primer incorporating a tail extension, allowing for detection based on fragment size.

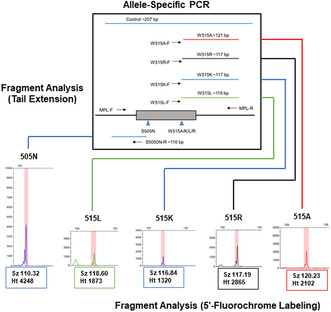

## 1 INTRODUCTION

Classification of myeloproliferative neoplasms (MPNs) includes hotspot testing of the *MPL* gene for essential thrombocythemia (ET) and primary myelofibrosis (PMF). *MPL* mutations are observed in approximately 10% of PMF and 3% of ET cases.^1^ The myeloproliferative leukemia virus oncogene (*MPL*) was originally identified in murine myeloproliferative leukemia virus models in 1990 as v‐*MPL*, followed by subsequent characterization of the human homolog (c‐*MPL*) 2 years later.^2^, ^3^ Located on chromosome 1p34, the 12‐exon *MPL* gene encodes for the 635 amino acid residue CD110/TPO‐R (Cluster of Differentiation 110/Thrombopoietin Receptor) transmembrane protein composed of an extracellular domain, a transmembrane domain, and a cytoplasmic domain.^4^ The binding of the MPL ligand thrombopoietin to the extracellular domain results in a dimeric rearrangement that activates two proteins from the Janus Kinase (JAK) family.^5^, ^6^, ^7^ These JAKs, JAK2, and TYK2, trigger activation of the downstream signaling pathways STAT (signal transducer and activator of transcription), MAPK/ERK (mitogen‐activated protein kinase, extracellular signal‐regulated kinase), and PI3K/AKT/mTOR (phosphatidylinositol‐3 kinase, serine/threonine–protein kinase, mechanistic target of rapamycin), ultimately playing a significant role in the regulation of cellular proliferation and differentiation.^8^, ^9^, ^10^, ^11^

The accelerated proliferation of myeloid lineage hematopoietic stem cells can result in various disorders, including myeloproliferative neoplasms (MPNs).^12^ This group of disorders is identified in the 2016 WHO classification of hematological malignancies and includes polycythemia vera (PV), primary myelofibrosis (PMF), and essential thrombocythemia (ET).^13^ One of the most well‐characterized mutations identified in MPNs is the p.V617F mutation in exon 14 of *JAK2*, found primarily in patients with PV.^14^, ^15^, ^16^ However, patients diagnosed with PMF and ET who lack the *JAK2* p.V617F mutation may display genetic mutations in *MPL* and/or *CALR* (calreticulin).^17^, ^18^, ^19^ These mutations can occur at low allelic fractions that conventional screening methods may miss. As a result, sensitive genetic testing capable of detecting low allelic variants is essential for identifying these mutations to diagnose and treat patients displaying symptoms of suspected MPNs.

*MPL* codon 515 mutations are detected in ~5%–10% of PMF patients and ~1% of ET patients.^17^ The most common mutations are p.W515L and p.W515K, while p.W515R and p.W515A are observed less frequently.^5^, ^20^ Additionally, the p.S505N mutation was characterized initially as constitutional in familial ET.^21^ However, the somatic mutation has been identified in ET and PMF, albeit in a small percentage of cases.^20^ Furthermore, while *MPL* codon 515 mutations help diagnose both ET and PMF, these mutations are also found in other disorders, including refractory anemia with ring sideroblasts associated with marked thrombocytosis (RARS‐T).^22^ These driver mutations in exon 10 of *MPL* result in a gain of function and constitutively activate downstream JAK/STAT signaling.^23^, ^24^ Accurate detection of *MPL* variants is essential in diagnosing ET and PMF. A multiplexed allele‐specific PCR approach in conjunction with fragment analysis allows for the design of a new assay built on a foundation of previously published studies.^25^, ^26^ This improved assay is simple and effective, enhancing diagnostic sensitivity, lowering hands‐on technician time, and limiting cost increases. Described here is the analysis and validation of a laboratory‐developed test (LDT) for detecting p.W515A/K/L/R and p.S505N mutations in *MPL*. The LDT was contrived from two previously published assays but with modifications.^25^, ^26^ Allele‐specific PCR was employed with subsequent fragment analysis to identify the W515A/K/L/R and S505N variants. Detection of a codon 515 or p.S505N variant is clinically significant for supporting a diagnosis of ET or PMF in the presence of specific symptoms and clinical features in conjunction with the absence of *JAK2* p.V617F.

## 2 MATERIALS AND METHODS

### 2.1 Nucleic acid extraction

Genomic DNA was isolated and extracted from 200 μL bone marrow aspirates or peripheral blood specimens using the EZ1 Advanced XL with the EZ1 DNA Blood Kit (Qiagen, Hilden, Germany). Following nucleic acid extraction, the 50 μL gDNA eluate was stored at −20°C.

### 2.2 Primer design

Primer sequence design and 5′‐fluorochrome labeling were based on previous studies using the MANE transcript, GenBank accession number NM_005373.3 (Thermo Fisher, Waltham, MA)^25^, ^26^ (Table 1). Primers designed for the *MPL* LDT included MPL‐F (5′‐TGGGCCGAAGTCTGACCCTTT‐3′), MPL‐R (5′‐CAGAGCGAACCAAGAATGCCTGT‐3′), W515A‐F (5′‐GCCTGCTGCTGCTGAGGGC‐3′), W515K‐F (5′‐GCCTGCTGCTGCTGAGGAA‐3′), W515L‐F (5′‐GGCCTGCTGCTGCTGAGATT‐3′), W515R‐F (5′‐GCCTGCTGCTGCTGAAGCG‐3′), W515R2‐F (5′‐GCCTGCTGCTGCTGAAGAG‐3′), and S505N‐R (5′‐CAGGAAACAGCTATGACCCAGGCCCAGGACGGCGT‐3′). The 5′‐fluorochrome labeling included FAM labeling for the MPL‐F and W515K‐F primers (Ex. 494 nm, Em. 530 nm), PET labeling for the W515A‐F primer (Ex. 558 nm, Em. 595 nm), VIC labeling for the W515L‐F primer (Ex. 538 nm, Em. 554 nm), and NED labeling for the W515R‐F and W515R2‐F primers (Ex. 546 nm, Em. 575 nm).

**TABLE 1:** Primer sequences and product sizes for PCR reactions.

Name	Sequence
MPL‐F	5′‐FAM‐TGGGCCGAAGTCTGACCCTTT‐3′
MPL‐R	5′‐CAGAGCGAACCAAGAATGCCTGT‐3′
W515A‐F	5′‐PET‐GCCTGCTGCTGCTGAGG**GC**‐3′
W515K‐F	5′‐FAM‐GCCTGCTGCTGCTGAGG**AA**‐3′
W515L‐F	5′‐VIC‐GGCCTGCTGCTGCTGAG*A*T**T**‐3′
W515R‐F	5′‐NED‐GCCTGCTGCTGCTGA*A*G**C**G‐3′
W515R2‐F	5′‐NED‐GCCTGCTGCTGCTGA*A*G**A**G‐3′
S505N‐R	5′‐CAGGAAACAGCTATGACCCAGGCCCAGGACGGCG**T**‐3′

*Note*: Bold bases, allele‐specific bases; italic bases, engineered mismatches.

### 2.3 Mutation‐specific gBlocks

Custom‐synthesized, double‐stranded DNA gBlocks (IDT, Coralville, IA) were hydrated with TE buffer to a 10 ng/μL concentration with 0.1 mg/mL yeast tRNA (Thermo Fisher). All gBlocks were designed as 100% mutant for each *MPL* mutation they carry but neutral for other *MPL* mutations as base positions for each mutation all occur at the W515 codon.

### 2.4 *MPL* W515A mutation sequence

GCTGGC TGGATGAGGG CGGGGCTCCG GCCCGGGTGG GCCGAAGTCT GACCCTTTTT GTCTCCTAGC CTGGATCTCC TTGGTGACCG CTCTGCATCT AGTGCTGGGC CTCAGCGCCG TCCTGGGCCT GCTGCTGCTG AGG **GC** GCAGT TTCCTGCACA CTACAGGTAC CGCCCCCGCC AGGCAGGAGA CTGGCGGTGG ACCAGGTGGA GCCGAAGGCC TGTAAACAGG CATTCTTGGT TCGCTCTGTG ACCCCAGATC TCCGTCCACC GCCCGTGCGC ACCTACGGCT TCGCCACTTC CTGCACGTCA CCTCTGGGAC TCGCCGCGGC TCCTTACACT CTAACACGCC.

### 2.5 *MPL* W515K mutation sequence

GCTGGC TGGATGAGGG CGGGGCTCCG GCCCGGGTGG GCCGAAGTCT GACCCTTTTT GTCTCCTAGC CTGGATCTCC TTGGTGACCG CTCTGCATCT AGTGCTGGGC CTCAGCGCCG TCCTGGGCCT GCTGCTGCTG AGG **AA** GCAGT TTCCTGCACA CTACAGGTAC CGCCCCCGCC AGGCAGGAGA CTGGCGGTGG ACCAGGTGGA GCCGAAGGCC TGTAAACAGG CATTCTTGGT TCGCTCTGTG ACCCCAGATC TCCGTCCACC GCCCGTGCGC ACCTACGGCT TCGCCACTTC CTGCACGTCA CCTCTGGGAC TCGCCGCGGC TCCTTACACT CTAACACGCC.

### 2.6 *MPL* W515L mutation sequence

GCTGGC TGGATGAGGG CGGGGCTCCG GCCCGGGTGG GCCGAAGTCT GACCCTTTTT GTCTCCTAGC CTGGATCTCC TTGGTGACCG CTCTGCATCT AGTGCTGGGC CTCAGCGCCG TCCTGGGCCT GCTGCTGCTG AGGT **T** GCAGT TTCCTGCACA CTACAGGTAC CGCCCCCGCC AGGCAGGAGA CTGGCGGTGG ACCAGGTGGA GCCGAAGGCC TGTAAACAGG CATTCTTGGT TCGCTCTGTG ACCCCAGATC TCCGTCCACC GCCCGTGCGC ACCTACGGCT TCGCCACTTC CTGCACGTCA CCTCTGGGAC TCGCCGCGGC TCCTTACACT CTAACACGCC.

### 2.7 *MPL* S505N mutation sequence

GCTGGC TGGATGAGGG CGGGGCTCCG GCCCGGGTGG GCCGAAGTCT GACCCTTTTT GTCTCCTAGC CTGGATCTCC TTGGTGACCG CTCTGCATCT AGTGCTGGGC CTCA **A** CGCCG TCCTGGGCCT GCTGCTGCTG AGGTGGCAGT TTCCTGCACA CTACAGGTAC CGCCCCCGCC AGGCAGGAGA CTGGCGGTGG ACCAGGTGGA GCCGAAGGCC TGTAAACAGG CATTCTTGGT TCGCTCTGTG ACCCCAGATC TCCGTCCACC GCCCGTGCGC ACCTACGGCT TCGCCACTTC CTGCACGTCA CCTCTGGGAC TCGCCGCGGC TCCTTACACT CTAACACGCC.

### 2.8 PCR

**FIGURE 1:**
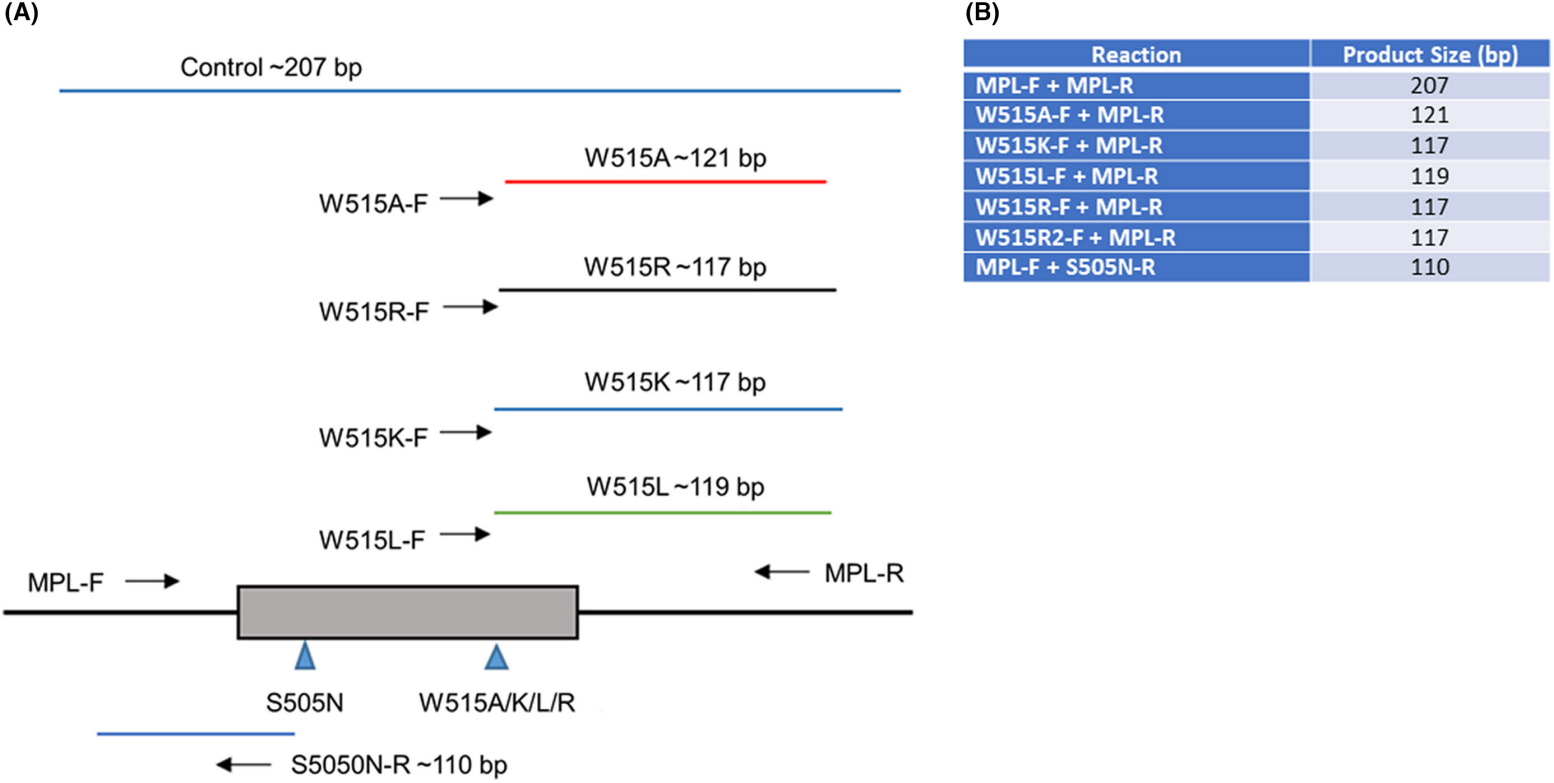
The *MPL* LDT utilizes multiple fluorochrome‐labeled primers to detect mutations at codons 515 and 505. (A) A total of seven primers are used for codon 515 mutation detection. These include five allele‐specific primers labeled with a 5′‐fluorochrome and two non‐allele‐specific primers for amplification of a 207 bp fragment of *MPL* exon 10, serving as a control for amplification in the absence of mutant allele detection. The codon 505 amplification reaction uses three primers: the FAM‐labeled *MPL*‐F, an unlabeled *MPL*‐R in conjunction, and an unlabeled, allele‐specific reverse primer for S505N mutation detection. (FAM: Blue, VIC: Green, NED: Black, PET: Red). (B) Seven PCR reactions are performed with products of various sizes to identify W515 and p.S505N variants. The products range in size from 110 to 207 bp.

Amplification of a 207 bp product encompassing the entirety of the *MPL* exon 10 sequence was performed using MPL‐F and MPL‐R as the forward and reverse primers (Figures 1A and S1). The W515A/K/L/R mutation products were amplified using the mutation‐specific forward primers in conjunction with the MPL‐R primer, generating amplicons of 121, 117, 119, and 117 bp, respectively. A 110 bp fragment was amplified for the S505N mutation using MPL‐F as the forward primer and S505N‐R as the reverse primer (Figure 1B).

Each W515 PCR reaction was 25 μL total and composed of 12.5 μL AmpliTaq Gold 360 Master Mix, 2 μL GC Enhancer, 5 μL W515 Primer Mix (120 nM MPL‐F, 240 nM W515L‐F, 240 nM K‐F, 160 nM W515R‐F, 160 nM W515R2‐F, 240 nm W515L‐F, 120 nM MPL‐F), 2.5 μL nuclease‐free H_2_O and 3 μL gDNA. The S505 PCR reaction was generated in the same volume and contained 12.5 μL AmpliTaq Gold 360, 5 μL S505N Primer Mix (120 nM MPL‐F, 240 nM S505N‐R, and 120 nM MPL‐R), 4.5 μL nuclease‐free H_2_O, and 3 μL gDNA (Supplementary Table S1).

PCR amplification of products was performed on a Veriti Dx thermal cycler (Thermo Fisher Scientific), beginning with an initial denaturation at 95°C for 10 min. This was followed by a 95°C denaturation for 30 s, a 61°C annealing process for 30 s, and an extension at 72°C for 1 min. The denaturation, annealing, and extension were repeated for 36 cycles, followed by a final extension at 72°C for 60 min and a completion incubation at 4°C. Two negative controls (one previously tested, W515A/K/L/R negative patient sample and one previously tested S505N negative patient sample), one 5% W515A/K/L/R composite positive control, one 10% S505N positive control, and two no‐template controls (NTC; one NTC for the codon 515 reaction and one NTC for the codon 505 reaction) were run in parallel for each set assay.

### 2.9 Fragment analysis

Following a 1:10 dilution of each amplicon and control, 12 μL of Hi‐Di formamide and 0.25 μL GeneScan 600 LIZ size ladder were combined and transferred to a 96‐well microplate. The plate was vortexed at 2000 rpm for 1–2 mins, followed by a quick spin to pool all reaction contents to the bottom of the wells. Reactions were denatured at 95°C in a thermal cycler for 3 mins., followed by incubation on ice for 4 mins. After a quick spin to ensure, all contents were retained at the bottom of the wells without bubbles, the plate was transferred to the ABI 3500 Genetic Analyzer for fragment analysis.

All patient information was incorporated into a macro‐enabled Excel (Microsoft) worksheet, generating an ABI 3500 run file for easy export, eliminating the need for manual data entry on the instrument (Figures S2 and S3). The ABI 3500 instrument protocol and size calling settings were optimized and adjusted for this assay (protocol available upon request). Upon run completion, interpretations of the data can be manually entered into the worksheet. GeneMapper 6 software was used to analyze all electrophoresis data. For each of the codon 515 mutations, patient samples with less than 500 RFUs and an internal control peak of >8000 RFUs are interpreted as negative. Patient samples with less than 2500 RFUs and an internal control peak of >8000 RFUs are interpreted as negative for the codon 505 mutation. Peak heights of ≥500 RFUs are interpreted as positive for codon 515 mutations.

## 3 RESULTS

Forty‐three patient samples were tested using the *MPL* LDT assay. All samples had previously tested negative for *JAK2* V617F using a qualitative LDT with a LoD of 1.0% and were reflexed for *MPL* analysis at a reference laboratory. The 43 patient samples included 38 peripheral blood samples and five bone marrow samples were collected in either EDTA or heparin. Based on reference laboratory results, 13 patient samples were positive for *MPL* mutations, and 30 samples had no mutation detected. The positive 13 patient samples included seven for the W515L mutation, two positives for the W515K mutation, two for the W515R mutation, and two positives for the S505N mutation. Using the *MPL* LDT, a concordance level of 100% was attained for patients positive for *MPL* mutations (13/13), and a concordance level of 100% was also achieved for patients negative for *MPL* mutations (30/30) (Table 2).

**TABLE 2:** Orthogonal testing demonstrates 100% concordance for all positive and negative patient samples.

Sample #	Sample type	W515 amplification results					S505N amplification results		Reference lab result
		W515A	W515K	W515L	W515R	IC	S505N	IC	
1	PB (EDTA)	Neg	Neg	Neg	Neg	Pos	Neg	Pos	No mutation detected
2	PB (EDTA)	Neg	Neg	Neg	Pos	Pos	Neg	Pos	W515R/S505C “Het”
3	PB (EDTA)	Neg	Pos	Neg	Neg	Pos	Neg	Pos	W515K “Het”
4	PB (EDTA)	Neg	Neg	Neg	Neg	Pos	Neg	Pos	No mutation detected
5	PB (EDTA)	Neg	Neg	Neg	Neg	Pos	Neg	Pos	No mutation detected
6	PB (EDTA)	SO peak	Neg	Pos	Neg	Pos	Neg	Pos	W515L “Het”
7	PB (EDTA)	Neg	Neg	Neg	Neg	Pos	Pos	Pos	S505N “Het”
8	PB (EDTA)	Neg	Neg	Neg	Neg	Pos	Neg	Pos	No mutation detected
9	PB (EDTA)	Neg	Neg	Neg	Neg	Pos	Neg	Pos	No mutation detected
10	PB (EDTA)	Neg	Neg	Neg	Neg	Pos	Pos	Pos	S505N 46%
11	PB (EDTA)	Neg	Neg	Neg	Neg	Pos	Neg	Pos	No mutation detected
12	BM (Hep)	Neg	Neg	Neg	Neg	Pos	Neg	Pos	No mutation detected
13	PB (EDTA)	SO peak	Neg	Pos	Neg	Pos	Neg	Pos	W515L detected
14	PB (EDTA)	Neg	Pos	SO peak	Neg	Pos	Neg	Pos	W515K “Het”
15	PB (EDTA)	Neg	Neg	Neg	Neg	Pos	Neg	Pos	No mutation detected
16	PB (EDTA)	Neg	Neg	Neg	Neg	Pos	Neg	Pos	No mutation detected
17	PB (EDTA)	Neg	Neg	Neg	Neg	Pos	Neg	Pos	No mutation detected
18	PB (EDTA)	Neg	Neg	Pos	Neg	Pos	Neg	Pos	W515L “Het”
19	PB (EDTA)	Neg	Neg	Neg	Neg	Pos	Neg	Pos	No mutation detected
20	PB (EDTA)	Neg	Neg	Neg	Neg	Pos	Neg	Pos	No mutation detected
21	PB (EDTA)	Neg	Neg	Neg	Neg	Pos	Neg	Pos	No mutation detected
22	PB (EDTA)	Neg	Neg	Pos	Neg	Pos	Neg	Pos	W515L detected
23	PB (EDTA)	Neg	Neg	Neg	Neg	Pos	Neg	Pos	No mutation detected
24	PB (EDTA)	Neg	Neg	Neg	Neg	Pos	Neg	Pos	No mutation detected
25	PB (EDTA)	Neg	Neg	Pos	Neg	Pos	Neg	Pos	W515L 3%
26	PB (EDTA)	Neg	Neg	Neg	Neg	Pos	Neg	Pos	No mutation detected
27	PB (EDTA)	Neg	Neg	Neg	Neg	Pos	Neg	Pos	No mutation detected
28	PB (EDTA)	Neg	Neg	Neg	Neg	Pos	Neg	Pos	No mutation detected
29	PB (EDTA)	Neg	Neg	Neg	Neg	Pos	Neg	Pos	No mutation detected
30	PB (EDTA)	Neg	Neg	Pos	Neg	Pos	Neg	Pos	W515L “Het”
31	PB (EDTA)	Neg	Neg	Neg	Neg	Pos	Neg	Pos	No mutation detected
32	PB (EDTA)	Neg	Neg	Neg	Neg	Pos	Neg	Pos	No mutation detected
33	PB (EDTA)	Neg	Neg	Neg	Pos	Pos	Neg	Pos	W515R 41%
34	PB (EDTA)	SO peak	Neg	Pos	Neg	Pos	Neg	Pos	W515L 70%
35	PB (EDTA)	Neg	Neg	Neg	Neg	Pos	Neg	Pos	No mutation detected
36	PB (EDTA)	Neg	Neg	Neg	Neg	Pos	Neg	Pos	No mutation detected
37	PB (EDTA)	Neg	Neg	Neg	Neg	Pos	Neg	Pos	No mutation detected
38	PB (EDTA)	Neg	Neg	Neg	Neg	Pos	Neg	Pos	No mutation detected
39	PB (EDTA)	Neg	Neg	Neg	Neg	Pos	Neg	Pos	No mutation detected
40	BM (Hep)	Neg	Neg	Neg	Neg	Pos	Neg	Pos	No mutation detected
41	BM (Hep)	Neg	Neg	Neg	Neg	Pos	Neg	Pos	No mutation detected
42	BM (Hep)	Neg	Neg	Neg	Neg	Pos	Neg	Pos	No mutation detected
43	BM (Hep)	Neg	Neg	Neg	Neg	Pos	Neg	Pos	No mutation detected

*Note*: SO stands for spectral overlap, whereby a peak was present in a different channel.

**FIGURE 2:**
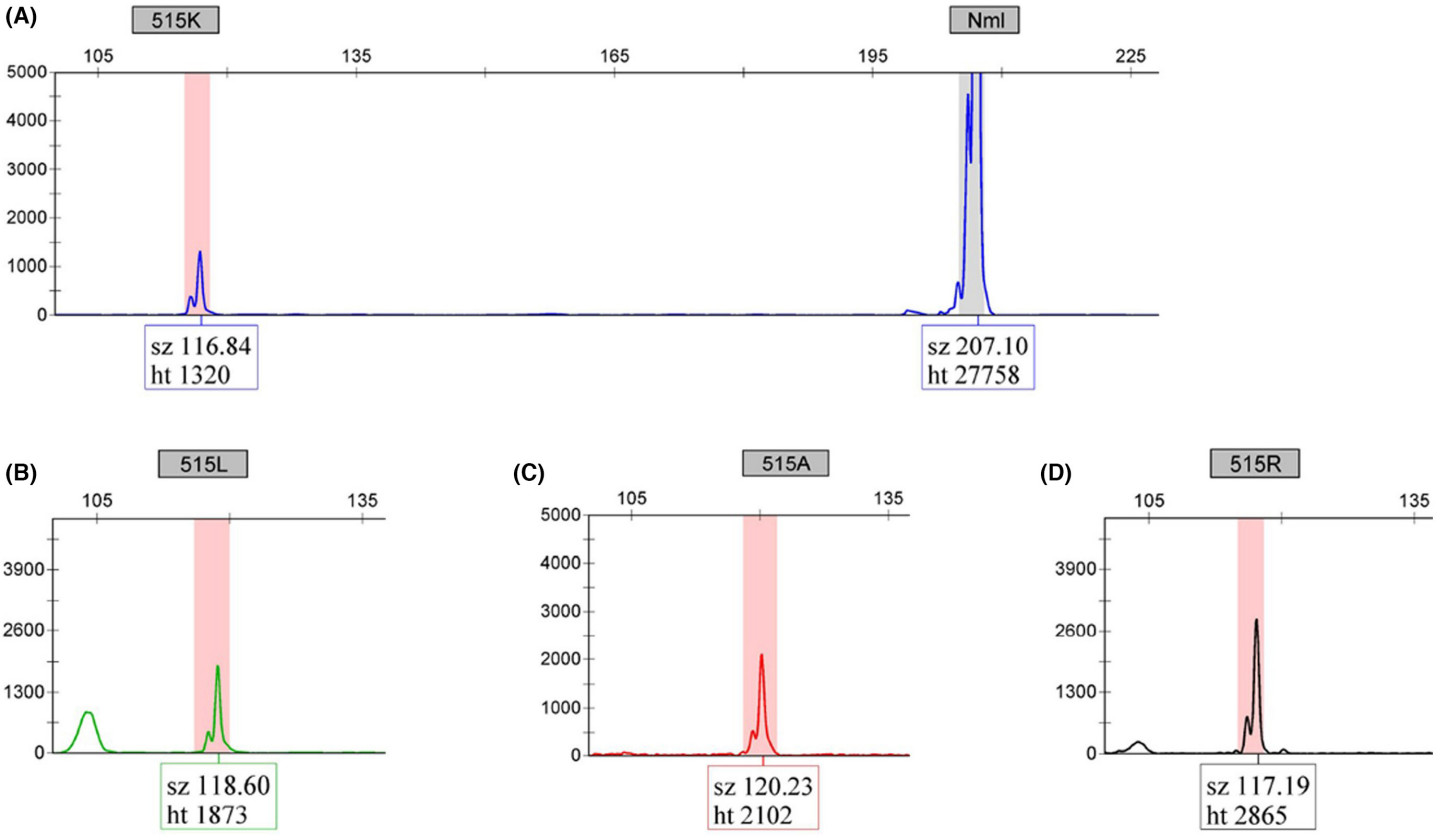
Fragments generated from allele‐specific PCR amplification are separated according to size and dye label via capillary electrophoresis on the ABI 3500 Genetic Analyzer. Peaks must have an RFU value ≥500 RFUs. The internal control (Nml) within the FAM panels must be detected with an RFU value ≥8000 RFUs. Each fluorochrome is associated with the specific mutations of codon 515: (A) FAM‐W515K, (B) VIC‐W515L, (C) NED‐W515R, and (D) PET‐W515A.

Since no patient samples harbored the W515A mutation, specificity for this mutation was established using a 356 bp synthetic DNA construct of *MPL* exon 10, which included the c.1543_1544delinsGC, p.W515A change. Additionally, synthetic DNAs were combined to construct a composite W515A/K/L/R positive/sensitivity control (Figure 2).

**FIGURE 3:**
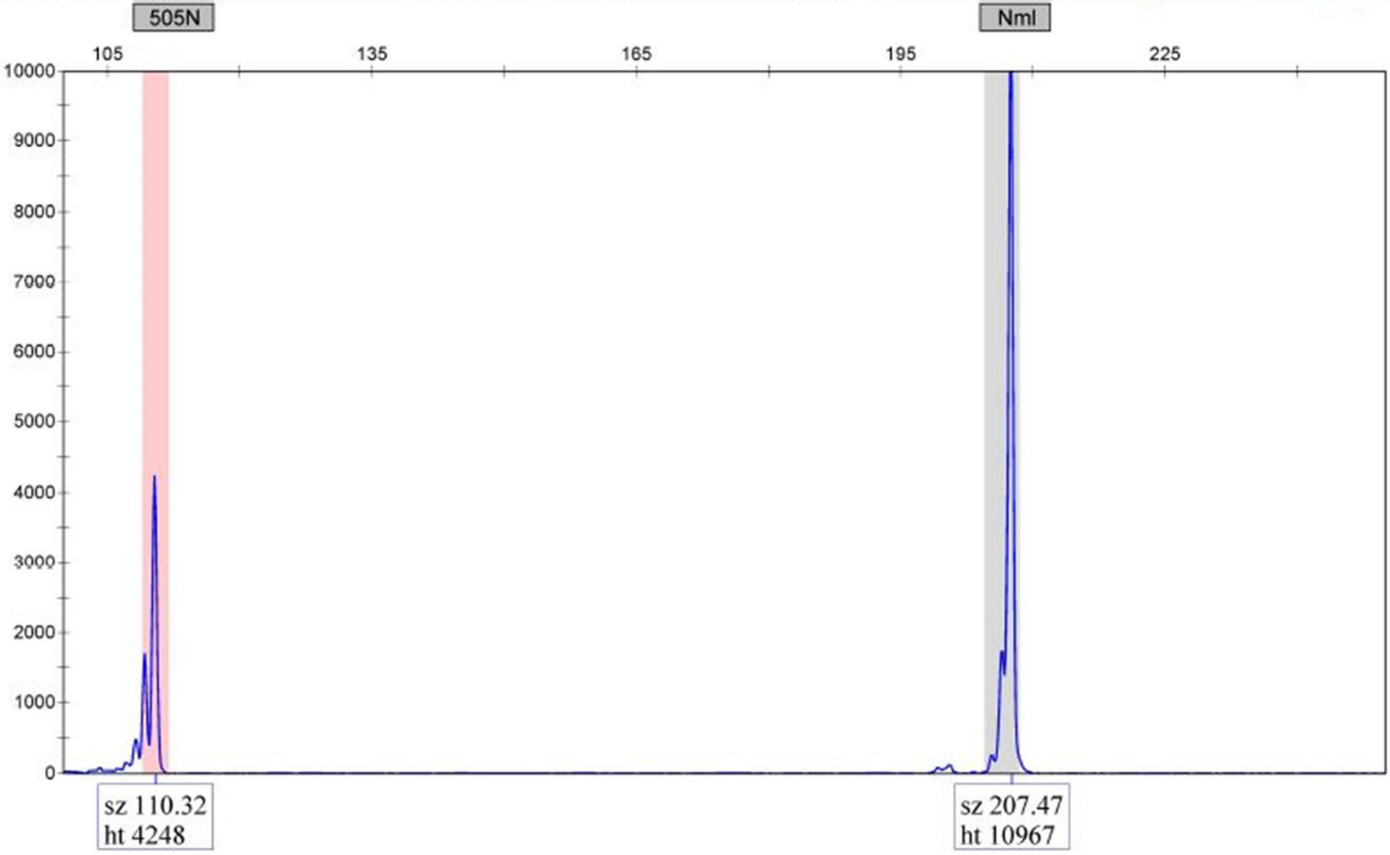
Fragment analysis of the codon 505 amplification product is completed using the GeneMapper software following capillary electrophoresis on the ABI 3500 genetic analyzer. S505N positive samples must have an RFU value of ≥2500 RFUs and an internal control peak (Nml) of ≥8000 RFUs.

A minimum peak height of ≥500 RFU was empirically determined as the threshold for interpreting a sample as positive for the W515 codon. However, the S505 codon requires a minimum peak height of ≥4000 RFU to be interpreted as positive (Figure 3). This results from the allele‐specific primer for the S505N mutation relying exclusively on a 3′ terminal T:C mismatch that inhibits the amplification of the normal allele. The weak mismatch permits a low level of amplification in the absence of an S505N mutant allele. During the validation, 69 S505N amplifications of expected negative samples were performed. A <1500 RFU background peak was observed in 58 of these amplifications, and 11 showed a background peak between 1500 and 2400 RFU. As a result of this higher background amplification, the LoD and RFU cutoff for a positive interpretation of the S505N mutation was set at a higher threshold than the W515 codon mutations.

The sensitivity of the *MPL* LDT was established with limits of 5% for W515 mutations and 10% for the S505N mutation. Positive patient samples and synthetic DNA constructs were utilized to establish the LoD for the *MPL* LDT and construct the positive controls. Only four of the 13 positive patient samples sent to reference laboratories included the mutation variant allele fraction (VAF). These included one W515R sample at 64.5% VAF, two W515L samples, one at 70% and another at 3%, and one S505N at 46% VAF. Mixing samples was performed to create dilutions. Combining a negative DNA sample with W515R and W515L samples (at 70%) were both diluted to 5% and 1% mutation VAF, while the W515L sample at 3% mutant was run undiluted. The S505N mutant was diluted in negative DNA to fractions of 10%, 5%, and 1% mutant. These samples were run in the *MPL* LDT in three separate runs with different thermal cyclers and ABI 3500 Genetic Analyzers (Figures 2 and 3; Tables 2 and 3).

**TABLE 3:** Sensitivity studies/determination of LoD.

Cycler	ABI 3500	5% mutant		1% mutant	
		Mutant peak height	IC peak height	Mutant peak height	IC peak height
W515R (41% VAF)					
E	R	2277	22,331	502	19,115
F	C	4297	31,277	900	29,365
N	C	1843	18,863	<500	15,812
W515L (70% VAF)					
E	R	11,451	23,388	2467	23,986
F	C	16,159	31,229	2648	28,584
N	C	8698	15,905	1806	15,541

W515L (3% VAF)			
Cycler	ABI3500	3% mutant	
		Mutant peak height	IC peak height
F	R	7171	30,025
N	C	7223	29,691
E	R	6375	26,784

S505N (46% VAF)							
Cycler	ABI3500	10% mutant		5% mutant		1% mutant	
		Mutant peak height	IC peak height	Mutant peak height	IC peak height	Mutant peak height	IC peak height
F	R	16,657	20,097	9011	18,227	4321	29,840
F	C	15,532	23,403	10,174	24,780	6157	30,813
N	C	9100	7273	6175	83,95	ND	ND

Inter‐run and intra‐run precision studies were performed using negative and positive patient samples. Runs were performed on different thermal cyclers and ABI 3500 Genetic Analyzers. Complete concordance was achieved for both negative (3/3) and positive samples (5/5) across three separate runs (Tables S2–S5).

## 4 DISCUSSION

Following a *JAK2* V617F‐negative result, reflex testing of *MPL* codons 515 and 505 is essential to molecular testing for Philadelphia chromosome‐negative MPNs. While *CALR* and *JAK2* exon 12–15 testing is included in this workup, identifying a mutation in *MPL* can readily facilitate the diagnosis of ET or PMF. The most common *MPL* MPN mutations listed in the COSMIC database (v97, release 29 November 22) include 18 reports of the p.S505N variant (COSM27286, COSM219103) and 564 reports for codon 515 variants. These include 405 reports of p.W515L (COSM24566, COSM18918), 23 reports of p.W515R (COSM29008, COSM41266, COSM43212), 12 reports of p.W515A (COSM27289), and 124 reports of p.W515K (COSM28487, COSM24567, COSM19193). Current *MPL* assays are predominantly focused on the W515L/K and S505N mutations. The *MPL* LDT described here allows for an all‐in‐one, expanded test using a multiplexed, allele‐specific PCR approach. Developing an LDT that simultaneously targets the p.S505N variant in addition to all four codon 515 variants, targeting the most common (such as p.W515L) and alternate (such as p.W515A) variants, increases the diagnostic yield. The development and validation of the *MPL* LDT allows for clinically sensitive detection (5% for mutations in codon 515 and 10% for mutations in codon 505) of the five most deleterious variants in the *MPL* gene using a single workflow. Additional variants outside of exon 10, where codons 515 and 505 reside, have been previously identified in other MPN cases. These include p.S204P/F and p.E230G, which occur in the extracellular domain, as well as p.Y591D/N and p.T119I, both located in the intracellular domain.^27^, ^28^ However, the rarity of these mutations, in addition to their lower gain‐of‐function activity compared with the p.W515L mutation does not make for standard routine testing of other exons in *MPL*.

Implementing an assay with increased diagnostic yield for *MPL* mutation testing is clinically useful as the annual incidence rates for ET and PMF in the United States are 1.55 per 100,000 person‐years and 0.44 per 100,000 person‐years, respectively.^29^ These incidence rates may be underreported due to a lack of testing in underserved populations, highlighting the importance of implementing a simple, cost‐effective test.

In this validation, 100% concordance was achieved for both negative (30/30) and positive patient samples (13/13). While the *MPL* LDT is validated with a LoD of 5% sensitivity for codon 515 variants and 10% for codon 505 variants, a patient sample harboring a 3% VAF p.W515L mutation was also detected. To ensure reproducibility and reporting consistency, we maintain the established LoD of 5% for all codon 515 variants, creating a more streamlined and efficient workflow. By optimizing and validating the *MPL* LDT to use allele‐specific PCR followed by fragment analysis on the ABI 3500 Genetic Analyzer, a sensitive and efficient assay can be employed for fast turnaround times. The in‐house designed Excel worksheet and associated VBA modules permit patient data and the run setup file to be readily exported. This file can then be imported to the ABI 3500 during run setup, eliminating transcription errors that may occur and providing a useful tool for laboratories lacking a fully integrated laboratory information management system (LIMS). Alternative methods for detecting low variant allele fractions, including quantitative or digital PCR, high‐resolution melting and next‐generation sequencing provide different strategies for laboratories striving for flexibility and integrating effective workflows while delivering clinically relevant results. Importantly, by testing for all variants simultaneously, this LDT provides increased diagnostic yield with little additional expense or hands‐on technician time.

## CONFLICT OF INTEREST STATEMENT

The authors declare no conflict of interest.

## Supporting information


Appendix S1.

## Data Availability

The data that support the findings of this study are available from the corresponding author upon reasonable request.
