# Supplementary material for: Expanded molecular detection of MPL codon p.W515 and p.S505N mutations in myeloproliferative neoplasms
Source: J Clin Lab Anal. 2023 Dec 7;37(23-24):e24992. doi: 10.1002/jcla.24992 (PMC10756946; doi:10.1002/jcla.24992)
Supplement: Supplementary file 1 — Appendix S1. [file JCLA-37-e24992-s001.zip › Figure_S3_Legend.docx]

**Figure S3. ABI3500 instrument and size calling protocols provide sufficient and appropriate properties for performing the *MPL* LDT.** Both the instrument and size calling protocols were exported from the ABI3500 and also included assay information, dye sets utilized, and file names.
