# Supplementary material for: Expanded molecular detection of MPL codon p.W515 and p.S505N mutations in myeloproliferative neoplasms
Source: J Clin Lab Anal. 2023 Dec 7;37(23-24):e24992. doi: 10.1002/jcla.24992 (PMC10756946; doi:10.1002/jcla.24992)
Supplement: Supplementary file 1 — Appendix S1. [file JCLA-37-e24992-s001.zip › Supp Figure_S2_Legend.docx]

**Figure S2. The MPL W515 and S505N worksheet provides a streamlined and efficient workflow for importing both patient data and the ABI3500 run file.** General run information, patient information, reagents utilized, and final interpretations are included in the first tab of the worksheet. This information is then automatically entered into the second tab, which displays the plate map, and the third tab, which is necessary for the final ABI3500 run file. With the appropriate VBA modules in place, the ABI3500 run file can be exported automatically by clicking on the “Export ABI 3500 Run File” button on the third tab of the worksheet.
