# Supplementary material for: Expanded molecular detection of MPL codon p.W515 and p.S505N mutations in myeloproliferative neoplasms
Source: J Clin Lab Anal. 2023 Dec 7;37(23-24):e24992. doi: 10.1002/jcla.24992 (PMC10756946; doi:10.1002/jcla.24992)
Supplement: Supplementary file 1 — Appendix S1. [file JCLA-37-e24992-s001.zip › Supplemental Figure 1 Legend.docx]

**Supplemental Figure 1. *MPL* exon 10 with mutation codons, including primer locations and gBlock.** Illustrated is the genomic sequence of the *MPL* exon 10 (grey line), its amino acid translation, and the location of codons 515 and 505 (purple blocks). Beveled blocks indicate the primer locations and 5’-3’ orientation, with the red beveled block identifying the multi-allele-specific primers for codon 515. The engineered gBlocks sit across the entire exon 10 and flanking genomic region (orange line). Sequence derived from GenBank reference NG_007525.
