# Supplementary material for: Expanded molecular detection of MPL codon p.W515 and p.S505N mutations in myeloproliferative neoplasms
Source: J Clin Lab Anal. 2023 Dec 7;37(23-24):e24992. doi: 10.1002/jcla.24992 (PMC10756946; doi:10.1002/jcla.24992)
Supplement: Supplementary file 1 — Appendix S1. [file JCLA-37-e24992-s001.zip › jcla24992-sup-0004-TablesS1-S5.docx]

**Supplementary Data**

**Supplementary Table 1. Stock and working concentrations of primers**

| **Volume (µL)** | **Component** | **Working Concentration** |
| --- | --- | --- |
| 9.0 | 100 µM MPL-F | 120 nM MPL-F |
| 18.0 | 100 µM W515L-F | 240 nM W515L-F |
| 18.0 | 100 µM W515K-F | 240 nM W515K-F |
| 12.0 | 100 µM W515R-F | 160 nM W515R-F |
| 12.0 | 100 µM W515R2-F | 160 nM W515R2-F |
| 18.0 | 100 µM W515A-F | 240 nM W515L-F |
| 9.0 | 100 µM MPL-R | 120 nM MPL-F |
| 1,404 | Nuclease-free H_2_O |  |

**Supplementary Table 2: Inter-run precision studies for mutation positive samples**

Patient 1: W515R/S505C Het (Peripheral Blood Sample)

| **Cycler** | **ABI 3500** | **W515R** | | **S505N** | |
| --- | --- | --- | --- | --- | --- |
|  |  | Mutant peaks | IC peak | Mutant peak | IC peak |
| E | R | R – 13,376 | 20,390 | < 1500 | 28,798 |
| N | C | R – 9,432 | 10,342 | < 1500 | 17,816 |
| F | R | R – 18,118 | 24,470 | < 1500 | 29,565 |

Patient 2: S505N at 46% VAF (Peripheral Blood Sample)

| **Cycler** | **ABI 3500** | **W515** | | **S505N** | |
| --- | --- | --- | --- | --- | --- |
|  |  | Mutant peaks | IC peak | Mutant peak | IC peak |
| F | C | < 500 | 20,305 | 14,188 | 8,286 |
| N | R | < 500 | 11,347 | 16,459 | 7,871 |
| F | C | < 500 | 19,720 | 19,344 | 19,344 |

Patient 3: W515K Het (Peripheral Blood Sample)

| **Cycler** | **ABI 3500** | **W515** | | **S505N** | |
| --- | --- | --- | --- | --- | --- |
|  |  | Mutant peaks | IC peak | Mutant peak | IC peak |
| F | C | K – 15,366 | 12,202 | < 1500 | 13,807 |
| N | R | K – 31,253  L – 5,597* | 15,268 | < 1500 | 15,000 |
| N | C | K – 28,317 | 22,666 | < 1500 | 28,585 |

Patient 4: W515L Het (Peripheral Blood Sample)

| **Cycler** | **ABI 3500** | **W515** | | **S505N** | |
| --- | --- | --- | --- | --- | --- |
|  |  | Mutant peaks | IC peak | Mutant peak | IC peak |
| N | C | L – 1,892 | 9,514 | < 1500 | 10,625 |
| N | C | L – 2,281 | 11,859 | < 1500 | 16,691 |
| F | R | L – 4,376 | 16,006 | < 1500 | 16,391 |

Patient 5: W515L at 3% VAF (Peripheral Blood Sample)

| **Cycler** | **ABI 3500** | **W515** | | **S505N** | |
| --- | --- | --- | --- | --- | --- |
|  |  | Mutant peaks | IC peak | Mutant peak | IC peak |
| F | R | L – 7,171 | 30,025 | < 2500 | 30,495 |
| N | C | L – 7,223 | 29,691 | < 2500 | 29,354 |
| E | R | L – 6,375 | 26,784 | ND | ND |

*** = Spectral overlap observed**

**Supplementary Table 3: Intra-run precision studies for negative patient samples**

Patient 1 (Bone Marrow Sample)

| **Cycler** | **ABI 3500** | **W515** | | **S505N** | |
| --- | --- | --- | --- | --- | --- |
|  |  | Mutant peaks | IC peak | Mutant peak | IC peak |
| N | C | < 500 | 18,794 | < 1500 | 22,226 |
|  |  | < 500 | 18,255 | < 1500 | 13,444 |
|  |  | < 500 | 16,830 | < 1500 | 21,892 |

Patient 2 (Peripheral Blood Sample)

| **Cycler** | **ABI 3500** | **W515** | | **S505N** | |
| --- | --- | --- | --- | --- | --- |
|  |  | Mutant peaks | IC peak | Mutant peak | IC peak |
| F | C | < 500 | 25,167 | < 1500 | 22,575 |
|  |  | < 500 | 16,898 | < 1500 | 24.062 |
|  |  | < 500 | 19,928 | < 1500 | 25,134 |

Patient 3 (Peripheral Blood Sample)

| **Cycler** | **ABI 3500** | **W515** | | **S505N** | |
| --- | --- | --- | --- | --- | --- |
|  |  | Mutant peaks | IC peak | Mutant peak | IC peak |
| E | R | < 500 | 27,611 | < 2500 | 31,551 |
|  |  | < 500 | 19,722 | < 1500 | 30,157 |
|  |  | < 500 | 27,980 | < 2500 | 31,077 |

**Supplementary Table 4:** **Intra-run precision studies for mutation positive samples**

Patient 1: W515R/S505C Het (Peripheral Blood Sample)

| **Cycler** | **ABI 3500** | **W515R** | | **S505N** | |
| --- | --- | --- | --- | --- | --- |
|  |  | Mutant peaks | IC peak | Mutant peak | IC peak |
| N | C | R – 9,454 | 10,305 | < 1500 | 14,395 |
|  |  | R – 12,094 | 13,273 | < 1500 | 17,859 |
|  |  | R – 6,747 | 7,449 | < 1500 | 21,194 |

Patient 2: W515K Het (Peripheral Blood Sample)

| **Cycler** | **ABI 3500** | **W515K/L** | | **S505N** | |
| --- | --- | --- | --- | --- | --- |
|  |  | Mutant peaks | IC peak | Mutant peak | IC peak |
| N | R | K – 31,457  L – 6,499* | 16, 127 | < 1500 | 14,756 |
|  |  | K – 30,747  L – 2,559* | 13,008 | < 1500 | 16,798 |
|  |  | K – 31,556  L – 7,733* | 16,668 | < 1500 | 13,448 |

Patient 3: W515L Het (Peripheral Blood Sample)

| **Cycler** | **ABI 3500** | **W515L** | | **S505N** | |
| --- | --- | --- | --- | --- | --- |
|  |  | Mutant peaks | IC peak | Mutant peak | IC peak |
| N | C | L – 1,918 | 10,299 | < 1500 | 17,994 |
|  |  | L – 3,033 | 15,603 | < 1500 | 17,635 |
|  |  | L – 1,891 | 9,674 | < 1500 | 14,444 |

**Supplementary Table 5: Inter-run precision studies for negative patient samples**

Patient 1 (Peripheral Blood Sample)

| **Cycler** | **ABI 3500** | **W515** | | **S505N** | |
| --- | --- | --- | --- | --- | --- |
|  |  | Mutant peaks | IC peak | Mutant peak | IC peak |
| E | R | < 500 | 22,882 | < 1500 | 27,636 |
| F | R | < 500 | 22,560 | < 1500 | 24,618 |
| N | C | < 500 | 21,963 | < 1500 | 24,865 |

Patient 2 (Bone Marrow Sample)

| **Cycler** | **ABI 3500** | **W515** | | **S505N** | |
| --- | --- | --- | --- | --- | --- |
|  |  | Mutant peaks | IC peak | Mutant peak | IC peak |
| F | C | < 500 | 22,328 | < 1500 | 25,133 |
| N | C | < 500 | 17,960 | < 1500 | 19,187 |
| E | R | < 500 | 27,942 | < 1500 | 25,738 |

Patient 3 (Peripheral Blood Sample)

| **Cycler** | **ABI 3500** | **W515** | | **S505N** | |
| --- | --- | --- | --- | --- | --- |
|  |  | Mutant peaks | IC peak | Mutant peak | IC peak |
| N | C | < 500 | 9,658 | < 1500 | 10,210 |
| F | R | < 500 | 24,623 | < 1500 | 28,430 |
| E | R | < 500 | 25,104 | < 2500 | 30,928 |
