# Supplementary figures and images for: Expanded molecular detection of MPL codon p.W515 and p.S505N mutations in myeloproliferative neoplasms
Source: J Clin Lab Anal. 2023 Dec 7;37(23-24):e24992. doi: 10.1002/jcla.24992 (PMC10756946; doi:10.1002/jcla.24992)

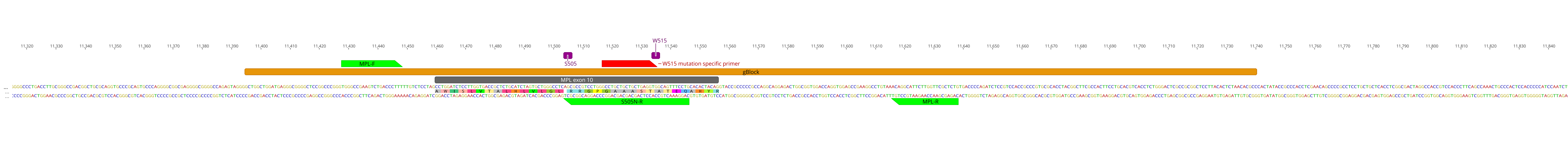

Supplement: Supplementary file 1 — Appendix S1. [file JCLA-37-e24992-s001.zip › jcla24992-sup-0001-FigureS1.pdf]
